# Quantifying the Implementation and Cost of a Multisite Antibiotic Stewardship Intervention for Asymptomatic Bacteriuria

**DOI:** 10.1017/ash.2023.198

**Published:** 2023-06-30

**Authors:** Eva Amenta, Larissa Grigoryan, Suja S. Rajan, David Ramsey, Jennifer R. Kramer, Annette Walder, Andrew Chou, John N. Van, Sarah L. Krein, Sylvia Hysong, Aanand D. Naik, Barbara W. Trautner

**Affiliations:** 1 Michael E. DeBakey Veteran Affairs Medical Center, Center for Innovations in Quality, Effectiveness, and Safety (IQuESt), Houston, TX, USA; 2 Section of Infectious Diseases, Department of Medicine, Baylor College of Medicine, Houston, TX, USA; 3 Department of Family and Community Medicine, Baylor College of Medicine, Houston, TX, USA; 4 UTHealth Science Center, Institute for Stroke and Cerebral Vascular Disease, Houston, TX, USA; 5 Section of Health Services Research, Department of Medicine, Baylor College of Medicine, Houston, TX, USA; 6 Center for Clinical Management Research, VA Ann Arbor Healthcare System, Ann Arbor, MI, USA; 7 Department of Medicine, University of Michigan, Ann Arbor, MI, USA; 8 Department of Management, Policy and Community Health, University of Texas School of Public Health, Houston, TX, USA; 9 UTHealth Consortium on Aging, University of Texas Health Science Center, Houston, TX, USA

## Abstract

**Objective:**

The intensity of an antibiotic stewardship intervention to achieve clinical impact is not known. We conducted a multisite dissemination project of an intervention to reduce treatment of asymptomatic bacteriuria (ASB) and studied: (1) the association between implementation metrics and clinical outcomes and (2) the cost of implementation.

**Design/Setting/Participants:**

A central site facilitated a multimodality intervention to decrease unnecessary urine cultures and antibiotic treatment in patients with ASB at 4 Veterans Affairs medical centers.

**Methods:**

The intervention consisted of a decision support aid algorithm and interactive teaching cases that provided in the moment audit and feedback on how to manage ASB. Implementation outcomes included minutes spent in intervention delivery, number of healthcare professionals reached, and number of sessions delivered. Clinical outcomes included days of antibiotic therapy (DOT), length of antibiotic therapy (LOT), and number of urine cultures ordered per 1000 bed days. Personnel reported weekly time logs.

**Results:**

Minutes spent in intervention delivery were inversely correlated with two clinical outcomes, DOT (*R* −0.3, *P* = .04) and LOT (*R* −0.3, *P* = .02). Number of healthcare professionals reached and number of sessions delivered were not correlated with clinical outcomes of DOT (*R* –0.003, *P* = .98, *R* = −0.059, *P* = .69) or LOT (*R* +0.073, *P* = .62, *R* −0.102, *P* = .49). Physician champions spent an average of 3.8% of effort on the intervention. The implementation cost was USD 22,299/year per site on average.

**Conclusions:**

The amount of time local teams spent in delivery of an antibiotic stewardship intervention was correlated with the desired decrease in antibiotic use. Implementing this successful antibiotic stewardship intervention required minimal time.

## Introduction

Antibiotic stewardship programs improve patient outcomes and have been required by the Centers for Medicare and Medicaid Services (CMS) as part of hospitals’ infection control and prevention departments since 2017.^1–3^ How to best implement evidence-based antibiotic stewardship interventions in hospitals and long-term care facilities is less clear. Measuring implementation and the cost of that effort are important to understand the full picture of an antibiotic stewardship intervention.

Implementation science is defined as “the scientific study of methods to promote the systematic uptake of proven clinical treatments, practices, organizational, and management interventions into routine practice, and hence to improve health.”^4^ Thus, implementation science helps to reveal what makes an intervention work or not work in practice.^5–7^ For example, if an antibiotic stewardship program falls short of its goals, was the intervention itself ineffective at changing behavior, or did the intervention site simply fail to carry out key aspects of the intervention? Proctor et al provide a conceptual framework for measuring the implementation process and define key factors that can be measured: acceptability, adoption, appropriateness, feasibility, fidelity, implementation cost, penetration, and sustainability.^8^ These factors encompass all stages of a project from early (eg, acceptability, appropriateness) to early/mid (eg, adoption, fidelity, and penetration) and late (eg, sustainability) implementation. However, metrics to determine implementation success are not standardized and are often not tied to clinically relevant outcomes.^8–11^

The cost of an implementation strategy, both in terms of money and time, is an important yet often neglected factor in implementation research and antibiotic stewardship. A budget impact analysis of the implementation cost can be used to estimate the amount a hospital or health system would need to invest to initiate as well as sustain an intervention.^12,13^ Antibiotic stewardship program (ASP) costs are relevant for hospital administration to understand and prioritize the investment needed for favorable clinical outcomes.^14^

Herein, we present our findings on defining and measuring clinically relevant implementation metrics and also implementation cost using a case example of an antibiotic stewardship intervention for asymptomatic bacteriuria (ASB).^15^ We conducted a hybrid effectiveness-implementation trial to disseminate a successful local antibiotic stewardship intervention to 4 sites. Urine cultures, days of antibiotic therapy (DOT), and length of antibiotic therapy (LOT) all decreased at intervention sites in comparison to contemporaneous control sites.^16,17^ Through this study, we also investigated 2 implementation questions.^11^ (1) We studied which measures of implementation would be related to the desired clinical outcomes. We hypothesized that the amount of time spent by the local site team delivering the intervention, the number of healthcare professionals reached at each site, and the number of intervention sessions delivered would be associated with fewer urine culture orders, fewer DOT, and shorter LOT, all standardized by 1000 bed days. (2) We also explored the cost of the intervention using micro-costing methods,^18^ specifically focusing on the time spent by each site’s team in delivering the intervention.

## Methods

A central coordinating site facilitated roll-out of an audit and feedback intervention to decrease unnecessary urine cultures and antibiotic treatment in patients with ASB. The intervention was implemented in 4 geographically distinct Veterans Affairs (VA) medical centers, all with the highest level of complexity (1a). Each site has an active antibiotic stewardship program in accordance with VHA Directive 1031, including physician and pharmacy champions. The focus of the intervention was on urine culture ordering and antibiotic use for ASB in acute medical wards and long-term care units. Four additional VA medical centers served as contemporaneous controls, to look at temporal trends in clinical outcomes during the intervention period. We did not have any on-site activity in the control sites, and they were not included in this analysis of implementation. The intervention occurred at site A for 16 months from February 4, 2019 to May 30, 2020, site B for 15 months from February 25, 2019 to May 30, 2020, site C for 12 months from May 10, 2019 to May 30, 2020, and site D for 11 months from June 20, 2019 to May 30, 2020.

### Antibiotic stewardship intervention

The project used an interrupted time series design with the 4 intervention sites supervised by a coordinating site comprising of a physician principal investigator (PI) and research coordinators. The research coordinators helped gather local data (surveys and chart reviews) and transmitted the information to the coordinating site. The central coordinating site provided external facilitation, which included organizing monthly meetings (with all sites and with each individual site’s team), providing site-specific teaching materials, collecting and analyzing data, and addressing logistical questions in real-time. The intervention had 2 major components: (1) a decision aid algorithm to help providers decide whether to send urine cultures or start antibiotics and (2) interactive educational sessions based on actual cases from participating sites that walked the participants through clinical decisions, in the context of learning to use our algorithm. Working through a case with the local site champion thus became a form of audit and feedback. The local site team members who delivered the educational sessions were ID physicians, ID pharmacists, or nurse practitioners. The targeted providers were medicine interns, residents, and attendings as well as nurse practitioners and physician assistants on acute medical and long-term care units. Each participating site had a physician site champion, a part-time research coordinator, and 1–2 additional participants (pharmacists and advanced practice providers). The focus of the intervention was on teaching providers in acute and long-term care to avoid ordering unnecessary urine cultures.

Implementation activities were measured and mapped to the implementation outcomes taxonomy from the Proctor Model.^8^ We measured the “dose” delivered of various implementation factors including the total time spent on the intervention (adoption), total number of healthcare professionals reached (penetration), and the total number of educational sessions (adoption) for each intervention site. The implementation activities were measured by intervention delivery log reports filled out by the person delivering the intervention (supplemental material). Time spent on the intervention was logged by all members of the local site team. Time spent on the intervention could include a number of activities, including responding to e-consults, leading teaching sessions, meeting with the coordinating site, meeting with local leadership, and helping develop teaching cases. Number of healthcare professionals reached was estimated by the person delivering the intervention at the intervention sites. For example, if the local site champion was giving grand rounds about this project, we asked them to visually count how many people were in the audience. In addition, the composition of the audience (physicians, nurses, pharmacists, etc) was noted. For number of educational sessions delivered, we asked site champions to note the type of activity, number of times, and venues in which they used our algorithm in the context of a clinical case for teaching purposes.

The clinical outcomes measured included LOT, DOT, and urine cultures obtained per 1000 bed days for each intervention site. Thousand bed days included all patients admitted to the study wards (acute and long-term care units). We captured all systemic antibiotics prescribed within 1 day prior or 2 days after a urine culture order as potentially related to ASB. Baseline measurements were obtained for the 12 months prior to the intervention start date from VA’s corporate data warehouse (CDW) within the VA Informatics and Computing Infrastructure (VINCI) environment.^19^ Clinical data were aggregated from monthly measurements from the intervention start date. Days of therapy refers to the aggregate total days for which any amount of an antibiotic was documented as administered to a patient. For example, if a patient received 5 days of 2 different antibiotics, DOT would be 10. Length of therapy refers to the number of days for which antibiotics were documented as administered to a patient. In the above example, LOT would be 5.

Clinical outcomes were reported previously.^20^ In brief, there was a decrease in urine culture orders and antibiotic use associated with urine cultures at sites that received the intervention. The intervention involved the use of internal and external facilitation to implement diagnostic and antibiotic stewardship for asymptomatic bacteriuria. In contrast, urine culture orders and antibiotic use appeared to increase at a group of contemporaneous control sites.

### Statistical analysis

The overall correlations between the accumulated monthly totals of the 3 implementation measures (the total time spent on the intervention, total number of healthcare professionals reached, and the total number of educational sessions) and the monthly frequencies of the 3 clinical outcomes (LOT, DOT, and urine cultures ordered) were calculated using data from the post-intervention period for all 4 sites using the SAS 9.4 PROC MIXED procedure. This approach considers the repeated measurements of the variables at each site, using the method described by Hamlet et al for the SAS User’s Group.^21^

### Implementation cost

An economic evaluation involving a micro-costing method^18^ with weekly personnel time logs was used to collect the minutes associated with the intervention tasks. Personnel time costs were the only expenses associated with this intervention; hence, no data was collected with respect to resource purchases or utilization. Data was collected from participating personnel at the 4 sites and was organized by the coordinating site and percent full-time effort (FTE) and costs were computed. The personnel included the local site team (physician, pharmacist, nurse practitioner, and part-time research coordinator) and the team at the coordinating site (physician and 2 research coordinators). An average of 39% of weeks had some missing time log data. A week with missing time log data was defined as one or more personnel on the team not submitting a time log for that week. To address missing time log data, we imputed the site and personnel-specific average time for the missing weeks. We have provided the averages and a range from all available data (see Tables 3 and 4, for FTE and annual cost, respectively). Missing time log data was only relevant to the cost analysis; intervention delivery data was captured through a different measure. Salary information from the Bureau of Labor Statistics and Association of American Medical Colleges were used to compute costs from the time-log information.

## Results

### Implementation outcomes

**Figure 1.**
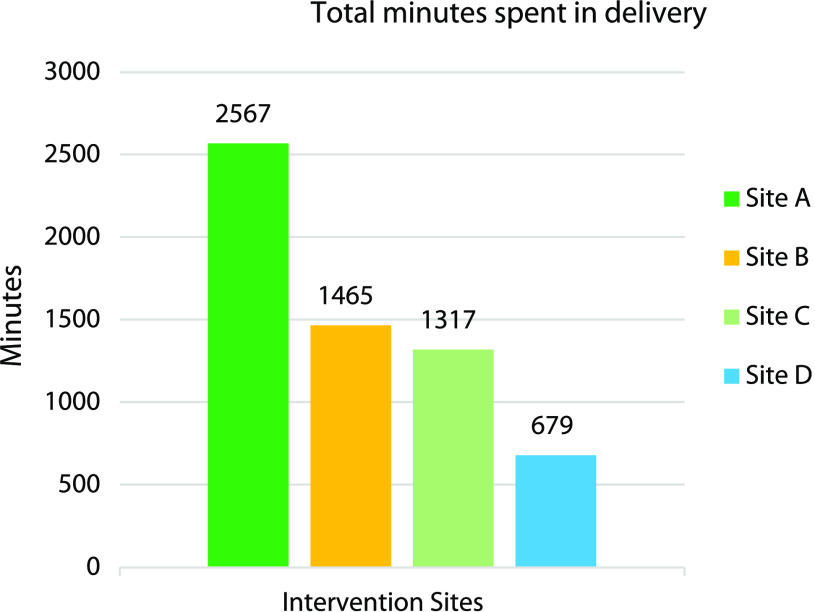
Total minutes spent in delivery of the intervention across 4 intervention sites.

Among the 4 intervention sites, the number of sessions, total time spent in content delivery, and total number of healthcare professionals reached varied widely (Table 1). Overall, the minutes spent in intervention delivery ranged from 679 minutes at the least engaged site to 2567 at the most engaged site (Figure 1). Minutes spent in delivery were inversely correlated with 2 of our 3 clinical metrics, DOT (*R* −0.3, *P* = .04) and LOT (*R* –0.3, *P* = .02); significance was not met for the correlation of minutes spent and urine cultures (*R* –0.24, *P* = .10) (Table 2). The number of healthcare professionals reached ranged from 433 to 798, and the number of sessions delivered ranged from 45 to 240. These implementation metrics did not have a significant relationship with clinical outcomes.

**Table 1.** Implementation Metrics of Intervention Across 4 Intervention Sites

Intervention Site	Dates of the Intervention (mo)	Total Number of Sessions	Total Minutes Spent in Delivery	Total Healthcare Providers Reached	Average of Delivery Time Per mo
Site A	16 mo	156 sessions	2567 min	643	160 min/mo
Site B	15 mo	240 sessions	1465 min	798	97 min/mo
Site C	12 mo	49 sessions	1317 min	542	109 min/mo
Site D	11 mo	45 sessions	679 min	433	61 min/mo

**Table 2.** Correlation Coefficients (with *P* values) Comparing Implementation Outcomes With Clinical Outcomes (Bolded Results are Statistically Significant)

	Total Number of Healthcare Professionals Reached	Number of Intervention Delivery Sessions	Minutes Spent in Delivery of the Intervention
Urine cultures	−0.003 (*P* = .99)	−0.075 (*P* = .61)	−0.241 (*P* = .10)
Days of therapy (DOT)	−0.003 (*P* = .98)	−0.059 (*P* = .69)	−**0.299 (** ***P*** **= .04)**
Length of therapy (LOT)	+0.073 (*P* = .62)	−0.102 (*P* = .49)	−**0.339 (** ***P*** **= .02)**

Educational activities were categorized as meeting with staff/in-services, e-consults/phone calls, educational activities with trainees and/or physicians, and grand rounds. Sites did not always report what type of activity occurred, but of the 254 specified educational sessions, 34% were educational activities with trainees and/or physicians, 62% were sessions with staff/in-services, 4% were e-consults/phone calls, and 0.7% were grand rounds.

### Implementation costs

Research coordinator time at the coordinating site and intervention sites comprised most of the personnel time, followed by the physician site champions (Table 3). Each intervention site required a mean of 10% (range 8.5–12.3) FTE/year of a research coordinator and 3.5% (range 2.9–4.3) FTE/year, 3.8% (range 1.1–6.3) FTE/year of a physician and pharmacist, respectively. The coordinating site required 37% FTE/year of a research coordinator and 9% FTE of a physician to spearhead the intervention. Physician champions predominantly spent their time delivering education and feedback, project coordination, and meetings. The implementation cost is USD 22,299/year per site on average (range 15,566–26,177) and USD 45,359/year for the coordinating site (Table 4).

**Table 3.** Percent full-time equivalent required per year for the intervention by professional by site

	Site A Average (min–max)	Site B Average (min–max)	Site C Average (min–max)	Site D Average (min–max)	Intervention Sites’ Average	Coordinating Site Average (min–max)
Infectious diseases physician	4.3	3.55	2.91	3.37	3.53	9.26
	1.52–12.52	1.97–6.64	1.25–6.10	1.56–8.82	1.58–8.52	5.68–34.58
Research coordinator	10.08	8.46	8.8	12.27	9.9	36.93
	5.08–22.19	5.26–20.01	5.99–27.07	5.98–26.74	5.58–24.00	29.17–47.92
Infectious diseases pharmacist	6.29	1.12	2.84	5	3.81	N/A
	1.00–47.22	0.03–1.98	0.91–7.03	0.13–8.90	0.52–16.28	
Nurse practitioner	N/A	N/A	N/A	1.37	1.37	N/A
				0.15–2.82	0.15–2.82	

Note. N/A, Not Applicable (this professional type was not on the local site’s team).

**Table 4.** Annual Cost by Site by Profession for the Intervention

	Site A	Site B	Site C	Site D	Average for Intervention Sites	Coordinating Site
Infectious diseases physician	10,495.15	8,676.23	7,099.25	8,238.35	8,627.25	22,607.67
	3,704.06–30,558.78	4,811.90–16,228.74	3,042.44–14,900.37	3,807.67–21,533.01	3,841.52–20,805.23	13,868.44–84,410.79
Research coordinator	6,209.41	5,208.66	5,421.63	7,557.85	6,099.39	22,751.51
	3,130.79–13,667.72	3,238.99–12,323.88	3,690.38–16,673.24	3,683.65–16,472.39	3,435.95–14,784.31	17,971.79–29,522.02
Infectious diseases pharmacist	9,472.52	1,681.55	4,272.69	7,530.43	5,739.30	0
	1,512.41–71,121.45	42.40–2,978.63	1,364.34–10,590.70	189.84–13,402.90	777.25–24,523.42	0.00–0.00
Nurse practitioner	0	0	0	1,832.62	1,832.62	0
	0.00–0.00	0.00–0.00	0.00–0.00	200.19–3,786.64	200.19–3,786.64	0.00–0.00
Total	26,177.08	15,566.44	16,793.56	25,159.25	22,298.55	45,359.18
	8,347.27–115,347.90	8,093.28–31,531.25	8,097.16–42,164.30	7,881.35–55,194.94	8,254.91–63,899.59	31,840.22–113,932.8

## Discussion

We found that the implementation metric of time spent in delivery of the intervention was inversely associated with 2 clinical outcomes, DOT and LOT. In other words, the more time spent at a given site in delivery of the intervention, the greater the reduction in antibiotic use. The cost of implementation was 22,299 USD/year for each site and 45,359 USD/year for the coordinating site, although the majority of the FTE from the coordinating site came from the research coordinator, whose role in local data collection and transmission would not be needed outside of a research study. For the other members of the local teams, implementation activities were designed to fit within their normal work activities (eg, team rounds), thus decreasing implementation-specific costs.

The need to translate research interventions into scalable, successful implementation projects has been an ongoing challenge within clinical research.^22–25^ Historically, implementation outcome studies do not explicitly link implementation factors to clinical outcomes. The effectiveness-implementation hybrid models designed by Curran et al address the need for studying both clinical effectiveness and implementation outcomes simultaneously. We used a hybrid effectiveness-implementation design to understand the effectiveness of implementing an ASB antibiotic stewardship intervention while also collecting clinically relevant outcomes associated with the intervention.

This project successfully delivered antibiotic stewardship for ASB in acute and long-term care facilities at 4 geographically diverse VA sites.^20^ Essential to its success was the effort put forth by the local site champions, measured by minutes spent in delivery of the intervention, number of sessions delivered, and number of healthcare providers reached. Importantly, one of our key implementation metrics (time spent in delivery) correlated with clinical effectiveness. This finding may be applicable to other antibiotic stewardship interventions and may be a useful tool to help plan and measure their implementation efforts.

Neither the number of participants reached nor the number of teaching/educational sessions correlated with clinical outcomes. We suspect that the amount of tailoring permitted by the local teams accounts for this finding, as local site teams conducted educational sessions in varied settings such as nursing fairs, grand rounds, and team rounds. The intensity of participants’ interaction or engagement with the intervention would have varied widely across these events. Thus, a single small group session of an infectious diseases physician with an internal medicine teaching team on rounds could have been more impactful than 3 grand rounds presentations to large but passive audiences or brief interactions with large numbers of individuals at a nursing fair.

Our participating sites reported limited resources available for antibiotic stewardship in preintervention surveys.^26^ We know from antibiotic stewardship resource studies more generally that the time required from physicians, pharmacists, and nurses is often more than what is supported financially by the institution.^14^ Per our cost analysis, the FTE required for the physician site champion to implement this intervention was 3.5% FTE (0.035). The main activity performed by the physician site champion was to deliver audit and feedback and to provide leadership for the local project. The time commitment by the central coordinating site was higher (36.93% FTE for research coordinator, 9.26% FTE for coordinating site physician), but this model lends itself well to additional scale-up to many local sites. Additional studies are needed to determine the ideal number of coordinating sites to local sites to maintain a sufficient level of engagement and support.

## Limitations

The implementation measures relied on self-recorded logs completed by individuals at the local sites. There may have been variability in how these were filled out between sites. In addition, we imputed missing data in the time logs by inserting time averages from weeks with complete data. The clinical outcomes of DOT and LOT were used as surrogates for antibiotic prescriptions for urinary infections and are objective, scalable, and reproducible. This study was not randomized, however, an interrupted time series methodology, the strongest quasi-experimental design, was employed. The goal for the intervention was to be carried out for at least 12 months but was stopped early due to COVID-19. No post-intervention data was collected to assess for sustainability of the intervention.

## Conclusion

Our antibiotic stewardship implementation study found that minutes spent in delivery of an antibiotic stewardship intervention were correlated with a decrease in antibiotic use (measured by DOT and LOT). This study provides direct evidence of an implementation metric linked to a desired clinical outcome. We also measured the cost of these efforts and found that implementation of the intervention required minimal time from the local physician champions. This suggests that the intervention can be scaled and disseminated and provides a possible framework for other antimicrobial stewardship interventions. We will continue to study the relationship between implementation and clinical outcomes as we scale up and disseminate this project more widely (AHRQ R18HS028776), while also exploring sustainability.

## Supplementary material

For supplementary material accompanying this paper visit http://doi.org/10.1017/ash.2023.198. click here to view supplementary material
