# Supplementary material for: Quantifying the Implementation and Cost of a Multisite Antibiotic Stewardship Intervention for Asymptomatic Bacteriuria
Source: Antimicrob Steward Healthc Epidemiol. 2023 Jun 30;3(1):e115. doi: 10.1017/ash.2023.198 (PMC10369447; doi:10.1017/ash.2023.198)
Supplement: Supplementary file 1 [file S2732494X23001985sup001.docx]

**Supplementary file 1:** Decision-aid for identifying symptomatic UTI vs. Asymptomatic Bacteriuria


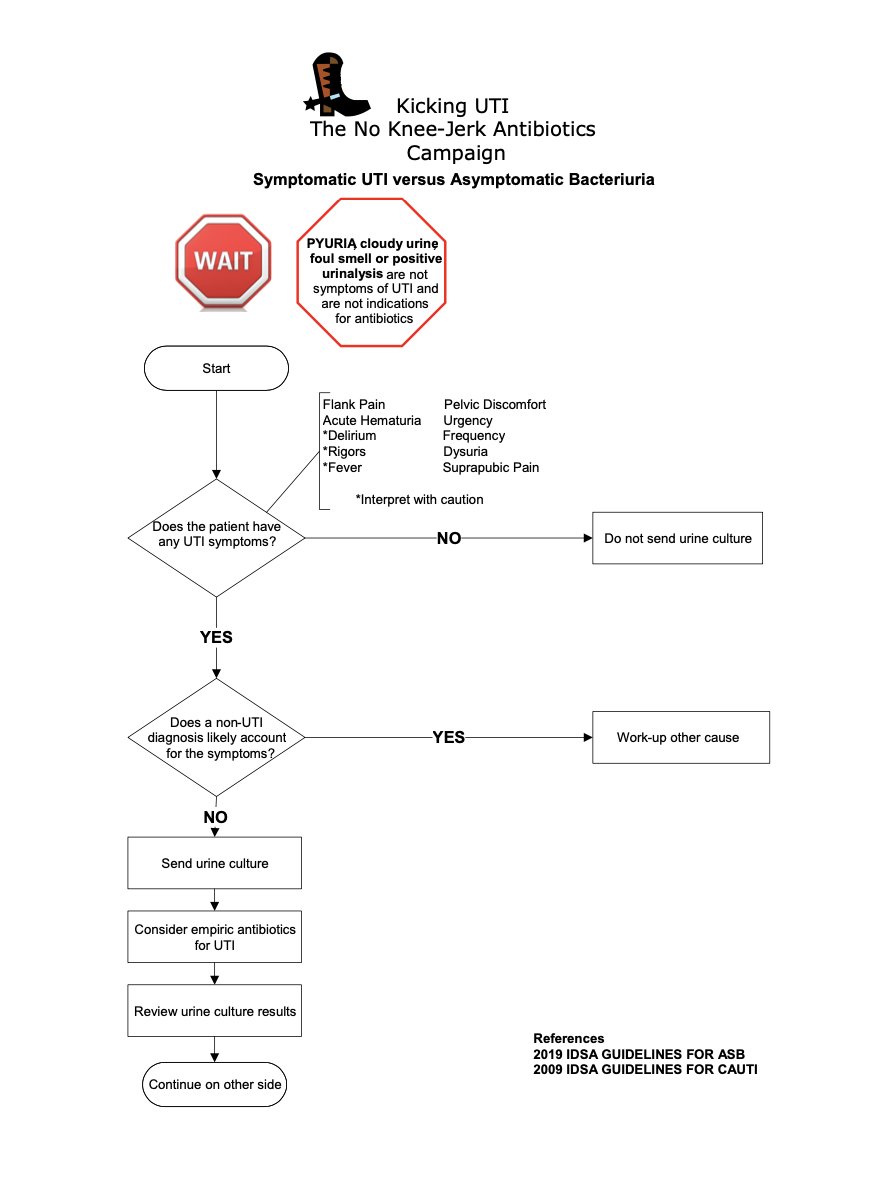


**
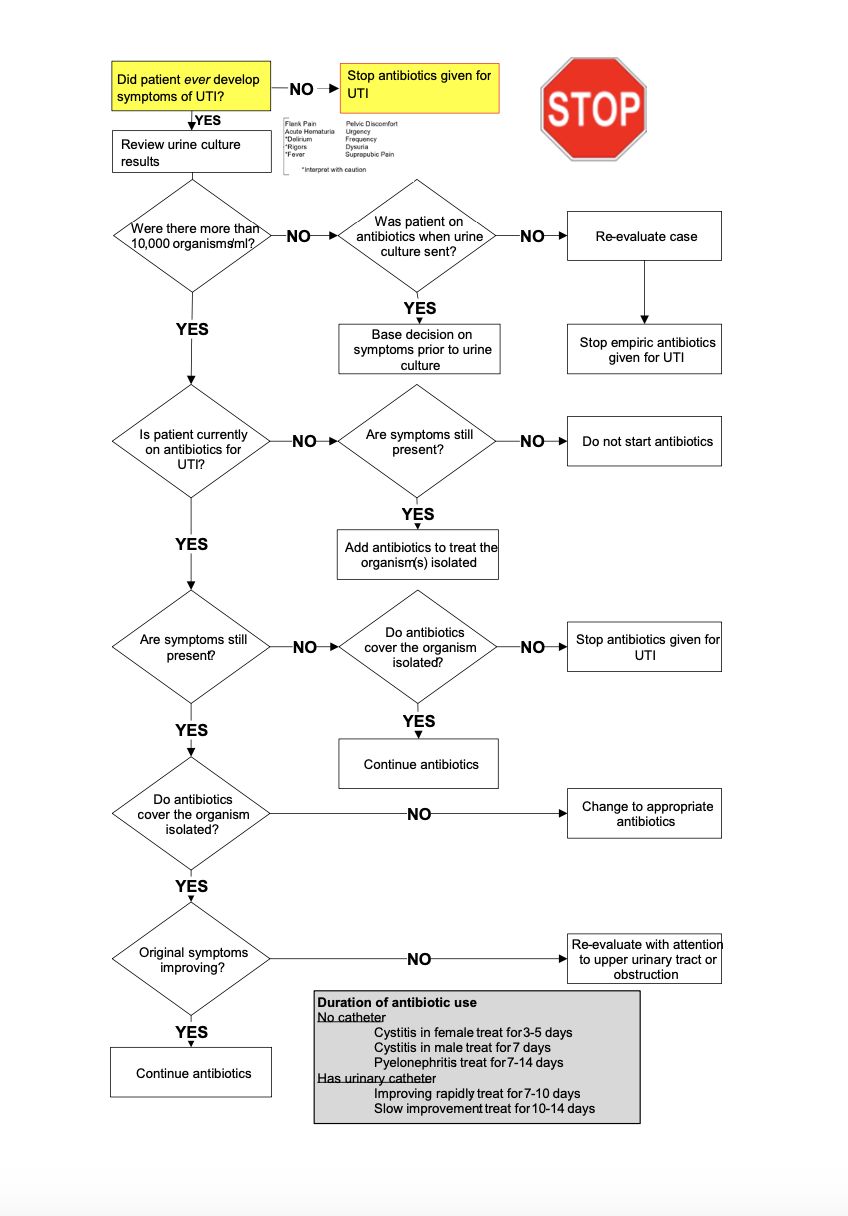
**

**Supplementary File 2:** Intervention Time Log

**Appendix E: Time Log**

Responder Initials: _______________

Site Name:__________________________________

Week of: :_______________

Please enter all time in minutes:

|  | Local Administration and Logistics | Intervention Delivery | Implementation Activities* |
| --- | --- | --- | --- |
| Monday |  |  |  |
| Tuesday |  |  |  |
| Wednesday |  |  |  |
| Thursday |  |  |  |
| Friday |  |  |  |
| Saturday |  |  |  |
| Sunday |  |  |  |

**Definitions:**

**Local Administration and Logistics:** Record any time you spent at your site in meetings regarding this project, on emails or project-related communications, creating awareness about the project in staff meetings, etc.

**Intervention Delivery:** Record how much time you spent delivering education or audit and feedback for the intervention. Any teaching setting is relevant—grand rounds, medicine team rounds, educational conferences, etc.

**Implementation Activities:** *These differ for the two arms. For the *virtual learning collaborative* arm, this definition will read: Implementation activities include time spent in training webinars, learning collaborative meetings, or office hours; time spent using the listserv; time spent accessing and reviewing project materials; time spent communicating with other sites in the project; and time spent working directly with the coordinating site.

For the *technical assistance* arm, this definition will read: Implementation activities include time spent in training webinars, time spent accessing and reviewing project materials, and time spent working directly with the coordinating site.

**Supplemental file 3:** Intervention Activity Log

**Appendix C: Intervention Activity Log**

**Your name:** _____________________

**Teaching Case Number presented (if used):** ___________________

**Type of activity:**

🞎 Grand Rounds 🞎 Teaching Conference 🞎 Team Rounds

🞎 In-service 🞎 E-consults 🞎 Staff Meetings 🞎 Phone Calls

🞎 Other (please describe: ____________________)

**Date of activity:** ____/____/_____

**Number of participants in attendance**:_____________

**Time spent in delivery in minutes:** _______________

**Composition of Audience:**

🞎 Staff physicians/providers 🞎 Residents in training 🞎 Infection Preventionists

🞎 Nurses 🞎 Certified Nursing Assistants

🞎 Pharmacists 🞎 Fellows

🞎 Other (please describe: ____________________)

**Comments**: __________________________________________________________________

____________________________________________________________________________
